# AGEs and Glucose Levels Modulate Type I and III Procollagen mRNA Synthesis in Dermal Fibroblasts Cells Culture

**DOI:** 10.1155/2008/473603

**Published:** 2008-04-03

**Authors:** Serban Iren Andreea, Costache Marieta, Dinischiotu Anca

**Affiliations:** ^1^Faculty of Veterinary Medicine, University of Agricultural Science and Veterinary Medicine, 105 Splaiul Independentei, 050097 Bucharest, Romania; ^2^Molecular Biology Center, Faculty of Biology, University of Bucharest, 91–95 Splaiul Independentei, 050095 Bucharest, Romania

## Abstract

In the dermis, fibroblasts play an important role in the turnover of the dermal extracellular matrix. Collagen I and III, the most important dermal proteins of the extracellular matrix, are progressively altered during ageing and diabetes. For mimicking diabetic conditions, the cultured human dermal fibroblasts were incubated with increasing amounts of AGE-modified BSA and *D*-glucose for 24 hours. The expression of procollagen *α*2(I) and procollagen *α*1(III) mRNA was analyzed by quantitative real-time PCR. Our data revealed that the treatment of fibroblasts with AGE-modified BSA upregulated the expression of procollagen *α*2(I) and procollagen *α*1(III) mRNA in a dose-dependent manner. High glucose levels mildly induced a profibrogenic pattern, increasing the procollagen *α*2(I) mRNA expression whereas there was a downregulation tendency of procollagen *α*1(III) mRNA.

## 1. INTRODUCTION

Elevated levels of blood glucose give birth to a vicious cycle of metabolic disturbances within the intracellular and extracellular environment and lead to a broad array of diabetes complications. In the hyperglycemic milieu activation of the aldose reductase pathway (AR), protein kinase C (PKC), especially the *β*-isoform, and generation of advanced glycation end products (AGEs) can be noticed [1]. In ageing, the nonenzymatic glycosylation of proteins or Maillard reaction occurs, which is a consequence of the elevated levels of glucose and is accelerated in diabetes [2]. The glycation reaction starts when the amino groups of a protein react nonenzymatically with glucose to forma Schiff base, stabilized through Amadori rearrangement, and it represents an early temporary step in the glycation process. In the advanced stage, complex reactions that lead to the formation of AGEs occur [3]. In ageing and diabetes, the AGEs levels of long life proteins of the extracellular matrix are increased [4]. These products cause cellular dysfunctions by multiple mechanisms, including receptor-independent and receptor-dependent processes, and can directly influence the structural integrity of the vessel wall and underlying basement membranes through excessive cross-linking of matrix molecules such as collagen and through disruption of matrix-matrix and matrix-cell interactions [5]. The formation of AGEs in skin collagen favors cross-linking reactions, resulting in decreased degradability and impaired dermal regeneration [6]. AGEs can bind to fibroblast cell membranes and may contribute to the progression of skin ageing [7].

The receptor for AGE (RAGE) is a multiligand receptor belonging to the immunoglobulin superfamily found in a widerange of cell types, including endothelial cells (EC), mononuclear phagocytes (MP), lymphocytes, vascular smooth muscle (VSMC), and neurons [8]. Recently, it was demonstrated that RAGE is highly expressed in skin and upregulated by AGEs and tumor necrosis factor-alpha (TNF-*α*) [9].

Collagen type I and III are the major structural components of the dermal extracellular matrix (ECM), representing over 70% and, respectively, 15% of skin dry weight and providing the dermis with tensile strength and stability [10]. Collagen metabolism is a complex process requiring a balance between synthesis and degradation through the action of cytokines and matrix metalloproteinase (MMPs). The most important profibrogenic cytokine is the transforming growth factor-beta (TGF-*β*1) [11].

The aim of this study was to investigate the influence of high glucose concentration and AGEs mimicking diabetic conditions on *procollagen 1 *α*2 (PCol 1 *α*2)* and *procollagen 3 *α*1 (PCol 3 *α*1)* genes expression in cultured human skin fibroblasts.

## 2. MATERIALS AND METHODS

### 2.1. Preparation and identification of AGE-modified BSA

Solutions of 1.6 M *D*-glucose (Sigma-Aldrich company) and 100 mg/mL bovine serum albumin (BSA Sigma-Aldrich, RIA grade, fraction *V*) have been coincubated insterile PBS 10 mM, pH 7.4 (Gibco) for 6 weeks under aerobic conditions at 37°C, in the presence of 3 mM NaN_3_ (Merck) to prevent bacterial growth [12]. Controls with BSA solution only were simultaneously kept under the same conditions. After 6 weeks, the free sugar was removed by dialysis against 10 mM PBS, pH 7.4 for 48 hours. The protein concentration (70 mg BSA/mL) was determined by Bradford method [13]. The formation of AGE-modified BSA (AGE-BSA) was analyzed by fast protein liquid chromatography (FPLC), SDS-PAGE, and by fluorescence spectroscopy.

**Fast protein liquid chromatography**

An FPLC automated system (*Ä*KTA FPLC-Amersham Pharmacia Biotech) with a size exclusion column (Superdex 200 HR 10/30) was used for separation of glycated and unglycated BSA. The samples of 1.55 *μ*g /*μ*L prepared in 10 mM PBS pH 7.4 were eluted in 5% acetonitrile (Merck) and 10 mM PBS pH 7.4, at a constant flow rate of 0.8 mL/min, and their absorption at 280 nm was automatically recorded.

**Gel electrophoresis**

AGE-BSA cross-linking and aggregation was investigated by 7.5% SDS-PAGE) with Mini Protean Bio-Rad equipment [14] and protein bands were stained by Coomassie Brilliant Blue R-250 (Sigma-Aldrich).

**AGE-BSA linked fluorescence assay**

The fluorescence detection of Maillard compounds was done using the parameter *λ*_ex_370 nm/*λ*_em_440 nm and for pentosidine-like products, *λ*_ex_335 nm/*λ*_em_385 nm was used. The fluorescence emission spectra between 380 and 600 nm (370 nm excitation) and between 350 and 500 nm (335 nm excitation) were scanned using aJASCO FP 750 spectrofluorometer [15].

### 2.2. Cell culture

Human dermal fibroblasts were obtained from skin biopsy sampled from the inferior pubian region of young normal female patients (average age 30 ± 2.3 years) by employing explants technique [16]. All patients gave informed written consent to tissue collection, which was conducted under a protocol approved by the Ethical Commission of National Institute of Endocrinology C.I. Parhon. The cells (2 × 10^4^/mL) were grown in DMEM (Sigma-Aldrich) medium supplemented with 10% fetal bovine serum (Sigma-Aldrich), sterile antimycotic solution 1X: penicillin 100 IU/mL, streptomycin 0.1 mg/mL and amphotericin 0.25 *μ*g/mL (Sigma-Aldrich), *D*-glucose 5.5 mM, 2% glutamine (Sigma-Aldrich), 0.22% NaHCO_3_ (Sigma-Aldrich), and 0.47% HEPES (Sigma-Aldrich) in a 5% CO_2_ humidified atmosphere, at 37°C.

### 2.3. Treatment of cells

The 70% confluent fibroblasts cultures (passage 3–5) were maintained 24 hours in the growth medium containing 0.5% fetal bovine serum for synchronization of cells cycle. Then they were treated with varying amounts of *D*-glucose: 5.5 mM (normoglycemic), 11 mM, 22 mM, and 33 mM (hyperglycemic) for 24 hours. The osmolarity of the medium was adjusted with *D*-mannitol after the addition of glucose, in order to have the same osmolarity in all samples. Other flasks of cells were treated with different amounts of sterile AGE-modified BSA, and BSA was added in order to adjust the total protein concentration to 5 mg/mL. The effect of *D*-glucose, *D*-mannitol, or AGE-BSA on cell viability was assessed by estimating the percent of cells excluding Trypan blue. There was no significant effect of *D*-glucose, *D*-mannitol, or AGE-BSA on cells viability. Over 95% of the cells excluded the dye.

### 2.4. Real-time PCR for collagen type I and III genes expression

RNA of treated cells was extracted using TRI Reagent kit (Sigma-Aldrich) according to the manufacturer’s recommendations [17] and by Chomezynski method [18]. RNA integrity and purity were electrophoretically verified by ethidium bromide staining and by OD_260_/OD_280_ nm absorption ratio. The specific sense and antisense oligonucleotide primers for target genes and for reference gene (Table 1) were designed with Beacon Designer programs (Premier Biosoft), based on the published genes sequences. 1 *μ*g aliquots of total RNA of each sample were reverse-transcribed into cDNA using Bio-Rad iScript cDNA Synthesis Kit, following the recommendations of the supplier. Real-time PCR was performed in the Bio-Rad iCycler iQ^TM^ in a final reaction mixture of 25 *μ*L consisting of 4 *μ*L diluted template cDNA, 12.5 Bio-Rad 2x iQ SYBER^TM^ Green Supermix, and 10 pmol of each forward and reverse primers (Applied Biosystems). The following real-time PCR experimental run protocol was used: denaturation program (95°C for 8 minutes), amplification, and quantification program repeated 45 times (95°C for 30 seconds, 54°C for 30 seconds, and 72°C for 30 seconds with a single fluorescence measurement), melting curve program repeated 80 times for 10 seconds (55°C–95°C with a heating rate of 0.5°C per second and a continuous florescence measurement). Melting curve analysis showed a single product for each transcript with melting temperatures as follows: for *PCol 1*α*2,* 89°C; for *PCol 3*α*1,* 91°C; and for *18 S RNA*, 89°C. In order to calculate the relative expression ratio (*R*) [19] of target genes (*PCol 1*α*2* and *PCol 3*α*1*) versus a reference gene, (*18 S RNA*) it was necessary to determine the crossing points (CPs) or cycle threshold (CT), the real-time PCR amplification efficiencies (Es), and the linearity for each transcript (CT is defined as the number of cycle at which the fluorescence signal is greater than a defined threshold in the logarithmic phase of amplification). Real-time PCR efficiencies were calculated from the given slope of a calibration curve CT = *f* (dilution series of cDNA for each gene) in iCycler iQ^TM^ software, according to the equation:

(1) $$E={\text{10}}^{\left(-1/\text{slope}\right)}\,,$$

see [20]. The investigated transcripts showed good real-time PCR efficiency rates for 1.93 *PCol 1*α*2*, 2.12 for *PCol 3*α*1*, and 1.89 for *18 S RNA* with high linearity (Pearson correlation coefficient *r* = 0.998). Generally, the relative expression ratio (*R*) of the target gene is calculated based on E and CT deviation of a sample versus a control and expressed in comparison with a reference gene according to Pfaffl equation [19]:

(2) $$R=\frac{{\left(E_{\text{target}}\right)}^{\Delta {\text{CT}}_{\text{target}}(\text{control-sample})}}{{\left(E_{\text{ref}}\right)}^{\Delta {\text{CT}}_{\text{ref}}\text{(control-sample)}}},$$

where *E*_target_ is the real-time PCR efficiency of target gene transcript, *E*_ref_ is the real-time PCR efficiency of a reference gene transcript, ΔCT_target_ is the CT deviation of control-sample of target gene transcript, and ΔCT_ref_ is the CT deviation of control-sample of reference gene transcript.

All experiments were done twice and samples were run in triplicate each time, the data having been expressed as the means ± standard deviation. The statistical significance of differences between the experiments was evaluated using Student’s t-test. *P* values < .05 were considered to be statistically significant.

## 3. RESULTS

### 3.1. Evidence of AGE-modified BSA formation

The AGEs content in the preparations was assessed by fluorescence measurements, SDS-PAGE analysis, and gel filtration studies.

**Fluorescence assays**

**Figure 1:**
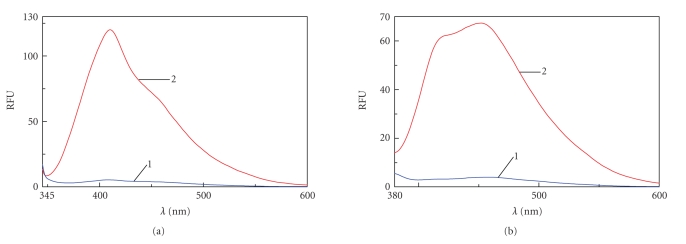
Fluorescence emission spectra in RFU (relative fluorescence units)/1 mg BSA; 6 weeks incubation of BSA (100 mg/mL) at 37°C in PBS 10 mM pH 7.4; curve (1) unglycated BSA (control); curve (2) BSA + 1.6 M *D*-glucose (AGE-modified BSA).(a) Fluorescence emission spectra of samples at 335 nm excitation. (b) Fluorescence emission spectra of samples at 370 nm excitation.

The fluorescence level measured at 385 nm emission wavelength after a 335 nm excitation wavelength was 3.77 fluorescence units (RFU) for control BSA and 74.9 RFU for AGE-BSA (Figure 1(a)). At 440 nm emission after a 370 nm excitation was 3.7 RFU for control BSA and 65.7 RFU for AGE-BSA. All fluorescence recorded was done at 1 mg/mL protein (Figure 1(b)).

**Gel electrophoresis**

**Figure 2:**
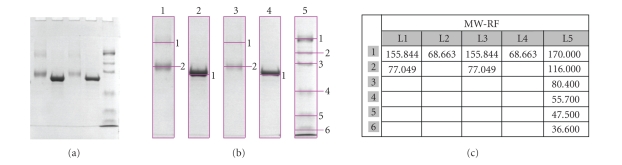
(a) and (b): SDS-PAGE electrophoretic profiles of 6 weeks glycation of BSA (100 mg/mL) at 37°C in PBS 10 mM pH 7.4; lanes 1 and 3: BSA + 1.6 *D*-glucose (AGE-modified BSA) (loaded with 10 *μ*g and 5 *μ*g protein, resp.); lanes 2 and 4: unglycated BSA (control) (loaded with 10 *μ*g and 5 *μ*g protein, resp.); lane 5: molecular weight marker (MWM 105 Bio-Rad). This was carried out using a 4% stacking and 7.5% resolving gel and Coomassie blue staining. (c) Corelation MW-RF.

SDS-PAGE analysis has shown the formation of an AGE-BSA monomer of 77.049 kDa and a 155.84 kDa dimer, whereas the monomer of control BSA was of 68.66 kDa (Figure 2).

**Chromatographic studies**

**Figure 3:**
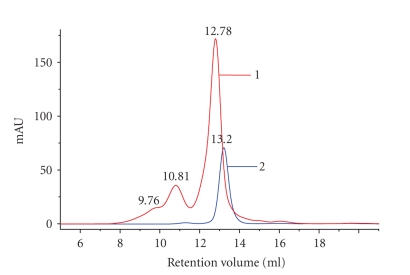
FPLC separation of glycated BSA on Superdex 200 HR 10/30 column, 155 *μ*g protein/100 *μ*L injection volumes: 6 weeks incubation of BSA (100 mg/mL) at 37°C in 10 mM PBS pH 7.4; curve (1) BSA + 1.6 M *D*-glucose (AGE-modified BSA); curve (2) unglycated BSA (control).

The FPLC elution pattern of BSA control sample showed only one peak corresponding to a 67.96 kDa molecular weight (retention volume 13.20 mL), while the glycated BSA (AGE-BSA) presented two peaks of 83.29 kDa (retention volume 12.78 mL) and 161.46 kDa (retention volume 10.81 mL). The peak with the retention volume at 12.78 mL showed a slightly increase in molecular weight and a major increase in 280 nm absorbance in comparison with the unglycated BSA peak. The peak with the retention volume 10.81 mL corresponds to a dimer with high molecular weight of 161.46 kDa of the glycated BSA monomer of 83.29 kDa (Figure 3). The increase in molecular weight of glycated BSA monomer and the formation of glycated BSA dimer with a higher molecular mass is probably due to the ability of AGEs compounds to generate intra- and intermolecular cross-linkings. Chromatographic data are in accordance with the SDS-PAGE results.

### 3.2. Influence of high glucose concentration on the expression of procollagen type I and III in cultured human dermal fibroblasts

**Figure 4:**
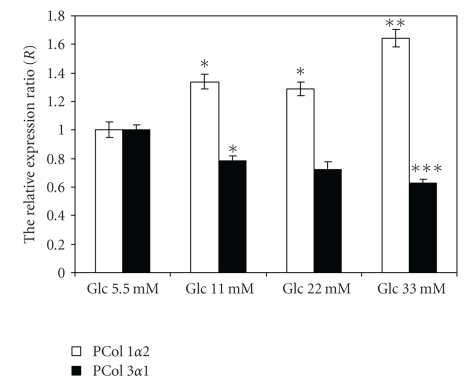
The relative expression ratios (*R*) for *procollagen 1α2* and for *procollagen 3α1* genes after 24 hours glucose treatment of culture human dermal fibroblasts. *R* was expressed in arbitrary units. The data are shown as the mean ± SD for two independent experiments run in triplicate each time with significant differences compared to control (5.5 mM glucose) at *P < .05, **P < .02, and ***P < .01.

The influence of high glucose concentration (mimicking diabetic conditions) on steady-state levels of the *procollagen 1 α2* and *procollagen 3 α1* mRNA was determined. Confluent monolayer fibroblasts were treated with 11 mM, 22 mM, and 33 mM *D*-glucose for 24 hours. The same type of cells treated with 5.5 mM *D*-glucose (normoglycemic conditions) was used as control. The mRNA expression was analyzed by quantitative real-time PCR relative to 18 S RNA. The high level in glucose compared to the control (5.5 mM glucose) resulted in a moderate increase of the relative expression ration (*R*) of procollagen *α*2(I) as follows: at 11 and at 22 mM glucose, the relative expression ratio (*R*) increased to 1.33 ± 0.051-fold (*P* < .05) and to 1.28 ± 0.048-fold (*P* < .05), respectively, and at 33 mM glucose the increase was 1.64 ± 0.063-fold *P* < .02. In the case of procollagen *α*1(III) mRNA at all concentration of glucose used in the cells treatment, there was a trend for downregulation compared to the control (Figure 4).

### 3.3. Effect of AGE-modified BSA on the expression of procollagen type I and III in cultured human dermal fibroblasts

**Figure 5:**
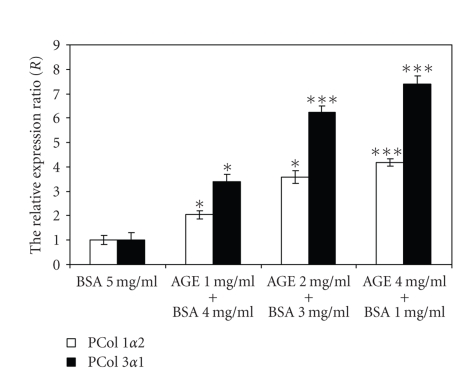
The relative expression ratios (*R*) for *procollagen 1α2* and for *procollagen 3α1* genes after 24 hours incubation with AGE-BSA of culture human dermal fibroblasts. *R* was expressed in arbitrary units. The data are shown as the mean ± SD for two independent experiments run in triplicate each time with significant differences compared to control (5 mg/mL BSA) at *P < .05 and ***P < .01.

Confluent monolayers human skin fibroblasts were exposed to increasing concentrations of AGE-modified BSA or BSA as control in culture medium containing 0.5% fetal bovine serum for 24 hours and mRNA expression for *procollagen1α2* and *procollagen3α1* was determined by real-time PCR. The treatment with increasing AGE-BSA levels adjusted to concentrations of 5 mg/mL protein with BSA upregulated the mRNA expression of procollagen *α*2(I) and procollagen *α*1(III) compared to the control (5 mg/mL BSA) (Figure 5). In response to 1 mg/mL of AGE-BSA, the relative expression ratio (*R*) for procollagen *α*2 (I) was 2.03 ± 0.15-fold (*P* < .05) and for procollagen *α*1(III) was 3.4 ± 0.3-fold (*P* < .05) on average. In the case of 2 mg/mL AGE-BSA treatment, the real upregulation ratio was, on average, 3.57 ± 0.26-fold (*P* < .05) and 6.22 ± 0.27 (*P* < 0.01) for procollagen *α*2 (I) and, respectively, for procollagen *α*1(III). The treatment of cultured dermal fibroblasts with 4 mg/mL AGE-BSA increased relative expression ratio (*R*) to 4.14 ± 0.14-fold (*P* < .01) and to 7.4 ± 0.32-fold (*P* < .01) for procollagen *α*2(I) and, respectively, for procollagen *α*1(III). These results showed that AGE-BSA upregulated the procollagen *α*1(III) mRNA expression to a great extent in comparison to procollagen *α*2(I) mRNA expression and this stimulation appeared to have a dose-dependent effect.

## 4. DISCUSSION

Tissue remodeling of extracellular matrix (ECM) is an essential and dynamic process associated with physiological responses and it involves the production and deposit of newly synthesized ECM components, as well as the degradation of ECM. The balance of these processes results in either preservation or alteration of the structure and functions of the support tissue [21]. Resorption of the ECM is mediated by MMPs, whereas generation of ECM is predominantly achieved through the production of collagen. Degradation of ECM generally characterizes pathological states such as arthritis or tumor invasion, whereas the increased generation of ECM underlies fibrotic diseases. Both processes are strictly regulated by complex networks of cellular and molecular interaction [22]. Mediators such as the profibrotic cytokines (TGF-*β*1, interleukins, and connective tissue growth factor-CTGF) released by resident cells, for examples, skin fibroblasts, or infiltrating leukocytes, monocytes, or macrophages may play a central role in the turnover of the dermal ECM. Expansion of ECM in fibrosis occurs in many tissues, including skin, as part of the end-organ complications in diabetes and chronic hyperglycemia. The formation of AGEs is considered as causative factor in diabetic tissue fibrosis. Elevated levels of blood glucose ignite a vicious cycle of metabolic disturbances with activation of multiple pathways. For example, it was demonstrated that AR is implicatedin the adverse cellular response to high levels of glucose [23]. Multiple studies indicate that expression and activity of AR are increased in experimental models and human tissuesin diabetes, including the diabetic kidney [24]. In addition to AR, isoforms of protein kinase C family, especially PKC^*β*^ isoform, has been associated with enhanced activity in hyperglycemia [25]. In vivo, pharmacologic blockade of PKC^*β*^ has been associated with improved vascular function in diabeticrats, as well as amelioration of accelerated mesangial expansion and expression of genes such as TGF-*β*1 and extracellular matrix components [11]. Our results suggest that high levels of glucose may influence the expansion of ECM and the process of skin aging through mild stimulation of *procollagen1α2* gene expression and possibly of other ECM proteins. A potential mediator for this effect is probablyTGF-*β*1, which is regulated by PKC^*β*^. Increased glucose flux through glycolysis and AR pathway leads to increased intracellular NADH/NAD^+^ ratio, which causes an inhibition of NAD^+^-dependent enzyme glyceraldehydes-3-phosphate dehydrogenase (GAPDH), which in turn will result in an increase in the dihydroxyacetone phosphate and glyceraldehyde-3-phosphate levels. These triosephosphates can be converted into PKC activator diacylglycerol, transformation accelerated by the increase in NADH/NAD^+^ ratio.

Another consequence of elevated levels of glucose is the Maillard reaction. In the advanced step, complex reactions that lead to the formation of AGEs occur [26]. Numerous studies have suggested a link among glucose-modified proteins, Amadori products, AGE, and activation of PKC^*β*^ isoforms. In cultured mesangial cells, inhibitors of PKC^*β*^ isoforms prevented the glycated albumin-induced increased expression of collagen IV [27]. Some in vivo studies showed that infusion of AGE-modified murine serumal bumin into nondiabetic mice for 4 weeks caused upregulation of glomerular *α*1(IV) collagen, laminin *β*1, and TGF-*β*1 transcriptionin the kidney [28].

In our experimental conditions, the AGE-modified BSA concentrations used appeared to be high, but the doses used in the treatment of cells (1, 2, and 4 mg/mL) represent the concentration of glycated protein not of AGEs products. By fluorescence, gel filtration chromatography, and SDS-PAGE assays, we have highlighted the formation of AGEs and the cross-linking of glycated BSA but we did not measure the level of AGEs compounds. On the other hand, fibroblasts can suffer in vivo directly from the effects of AGEs formed during the degradation of matrix proteins that have a long life and important amounts of these compounds can accumulate in time.

Finally, by means of real-time PCR, we revealed that AGE-modified BSA interacts with cultured human dermal fibroblasts and influence their function by significant upregulation in a dose-dependent manner of both *procollagen 1*α*2* and *procollagen 3α1* genes expression. In addition, at all AGE-BSA doses used for the cultured fibroblasts treatments, the ratio of procollagen *α*1(III)/procollagen *α*2(I) mRNA remained constant to approximately 1.7 on average.

This could probably alter the turnover of collagenous ECM in the skin and contribute to a decreased tensile strength and mechanical stability of connective tissues and a difficult healing in diabetes. Twigg et al. [29] have reported that in confluent monolayers of cultured human dermal fibroblasts, the connective tissue growth factor (CTGF) is upregulated at the mRNA and protein levels by AGEs. Later, they have shown that CTGF contributes significantly, in human dermal fibroblasts, to AGEs upregulation of fibronectin, another ECM component like collagen, through a PKC-dependent mechanism [30]. In the same year Okano et al. [7] demonstrated that AGEs which are accumulating in elastin, fibronectin, and collagens bind to fibroblast membranes at concentrations of 2.5–40 mg/mL. Our data are in accord with other studies which described AGEs increased collagen production in normal rat kidney fibroblasts [31]. Recently, Lohwasser et al. demonstrated for the first time that RAGE protein is highly expressed in human skin and in cultured human skin fibroblasts and AGE adduct upregulated RAGE expression and induced significantly upregulated expression of CTGF, TGF-*β*1, and procollagen *α*1(I) [9]. Also RAGE induction through AGE-BSA and TNF-*α* was shown before in human umbilical vein endothelial cells [32] and through AGE-BSA in normal rat kidney fibroblasts [31].

It seems that the most important ECM-related protein genes *Pcol 1*α*2* and *Pcol 3*α*1* are upregulated in the presence of AGEs and to a less extent by high levels of glucose in cultured human dermal fibroblast possibly by receptor-independent/dependent pathways.

## Figures and Tables

**Table 1:** Sequences of human primers used for real-time PCR.

Gene (mRNA)	Oligonucleotide primer sequence (5′ -3′)	Amplification fragment	Annealing temperature (°C)	
			Calculate	Use
*Procolagen 1 α2 (PCol 1α2)* sense	GTGGTTACTACTGGATTGACC	331	53.4	54
*Procolagen 1 α2 (PCol1α2)* antisense	TTGCCAGTCTCCTCATCCAT			
*Procolagen 3 α1 (Pcol3α1)* sense	GGAGTAGCAGTAGGAGGAC	91	54	54
*Procolagen 1 α1 (PCol3α1)* antisense	AACCAGGATGACCAGATGTA			
*18S RNA* sense	CTCAACACGGGAAACCTCAC	133	53.5	54
*18S RNA* antisense	TTATCGGAATTAACCAGACAAATCG			
